# Etymologia: Hemozoin

**DOI:** 10.3201/eid2202.ET2202

**Published:** 2016-02

**Keywords:** etymologia, hemozoin, pigment, hemoglobin, plasmodium, parasites, malaria

## Hemozoin [heʺmo-zoʹin]

From the Greek *haima* (“blood”) + *zoon* (“animal”), hemozoin (Figure) is a pigment produced by malaria parasites from hemoglobin in the host’s red blood cells. This pigment was first observed by Johann Heinrich Meckel in 1847 in the blood and spleen of a mentally impaired person. In 1849, Rudolf Virchow made the connection to malaria, but it was initially believed that it was produced in the patient’s spleen as a part of the immune response to malaria. In 1880, Charles Louis Alphonse Laveran observed pigmented parasites in the blood of an Algerian soldier and realized that the parasites, not the patient, produce “malaria pigment.” The term “hemozoin” was coined by Louis Westenra Sambon.

**Figure:**
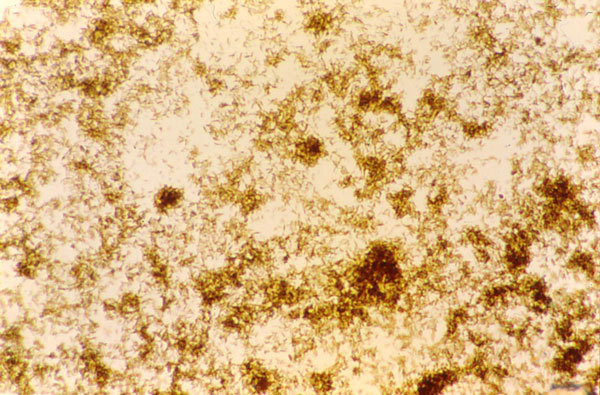
Hemozoin
